# Phosphoglucose isomerase directs the inflammatory response, calcium influx and fibroblast migration in keloids

**DOI:** 10.1080/20565623.2026.2615968

**Published:** 2026-01-20

**Authors:** Ying-Yi Lu, Chun-Ching Lu, Wei-Ting Wang, Chieh-Hsin Wu

**Affiliations:** a Department of Dermatology, Kaohsiung Veterans General Hospital, Kaohsiung, Taiwan; b School of Medicine, College of Medicine, National Sun Yat-sen University, Kaohsiung, Taiwan; c Department of Health and Beauty, Shu-Zen Junior College of Medicine and Management, Kaohsiung, Taiwan; d Department of Orthopaedics and Traumatology, National Yang Ming Chiao Tung University Hospital, Yilan, Taiwan; e Department of Orthopaedics, School of Medicine, National Yang Ming Chiao Tung University, Taipei, Taiwan; f Department of Radiology, Tri-Service General Hospital, National Defense Medical Center, Taipei City, Taiwan; g Division of Neurosurgery, Department of Surgery, Kaohsiung Medical University Hospital, Kaohsiung, Taiwan; h Department of Surgery, School of Medicine, College of Medicine, Kaohsiung Medical University, Kaohsiung, Taiwan; i Center for Big Data Research, Kaohsiung Medical University, Kaohsiung, Taiwan; j Drug Development and Value Creation Research Center, Kaohsiung Medical University, Kaohsiung, Taiwan

**Keywords:** Calcium, inflammation, fibroblasts, keloids, phosphoglucose isomerase

## Abstract

**Aim:**

Keloids are regarded as an inflammatory skin disease with altered metabolic demands. Calcium ions are known to regulate cell movement. Phosphoglucose isomerase (PGI) not only balances glucose metabolism but also acts as a multifunctional cytokine, as those calcium ions do. Here, for the first time, we aimed to explore the intracellular calcium level controlled by PGI in keloid fibroblasts (KFs) and normal fibroblasts (NFs). In addition, whether PGI regulates the biological functions of KFs via the inflammatory status was investigated.

**Methods:**

The inflammatory status, fibrotic activity, and migration ability of KFs and NFs were evaluated via RT–PCR, western blot analysis, and scratch assay. We inhibited PGI with erythrose 4-phosphate (ER4P) to determine whether PGI regulates KF migration.

**Results:**

The upregulation of PGI expression was measured in both KFs and keloid tissues. Suppressing PGI inhibited SMA and type I collagen expression, and cell migration in KFs. Indeed, PGI regulated inflammation and calcium influx in KFs.

**Conclusions:**

Our study is the first to show that PGI regulates the migration of KFs via a calcium influx-dependent inflammatory response and that blocking PGI might be a therapeutic strategy for keloids.

## ARTICLE HIGHLIGHTS

1. Keloids are a pathological scar with altered metabolic demands. With increasing capacity to synthesize collagen, keloid fibroblasts continuously have high metabolic demands. To overcome energetic consumption, keloid fibroblasts undergo metabolic reprogramming with reduced oxidative phosphorylation and augmented glycolysis.
2. Keloids are also regarded as an inflammatory skin disease. Excessive and prolonged inflammatory responses tend to lead to the development of pathological scars. The inflammation intensity is positively associated with the scar size.
3. Free calcium ions are important for cellular activities including cell proliferation, differentiation, movement and death.
4. Phosphoglucose isomerase (PGI) not only balances glucose metabolism but also acts as a multifunctional cytokine to induce cell proliferation, differentiation, or metastasis.
5. PGI expression was increased in hyperfibrotic regions in keloid tissues and keloid fibroblasts (KFs).
6. PGI controls fibrotic activity in KFs by regulating the synthesis of α-SMA, collagen I and the release of TGF-β.
7. PGI regulated inflammatory activity in KFs such as IL-33, NK-κB, TNF-α, IL-1 and IL-6.
8. Blocking PGI decreased enhanced cytosolic Ca2+ signaling in KFs.
9. Blocking PGI inhibits fibroblast migration in KFs.
10. Our study is the first to show that PGI regulates the migration of KFs via a calcium influx-dependent inflammatory response. Blocking PGI might be a therapeutic strategy for keloids.

## 1. Introduction

As proliferative scars, keloids appear as red, fixed, firm lesions with a shiny surface and beyond the original wound. Keloids are prone to occur in highly mobile regions with high tension [1–3]. They are often accompanied by intractable tenderness, pruritus, and even disfigurement [4]. More than half of patients have a family history of genetic susceptibility [5]. Keloids are unlikely to regress spontaneously and have a high recurrence rate, so that keloids can cause overwhelming distress [6,7]. Because the pathogenesis of keloids remains elusive, effective treatments still need investigation [7]. Multifactorial etiologies induce keloid formation, such as excessive fibroblast proliferation, myofibroblast overactivation, altered growth factor regulation, dysregulated mechanical forces, aberrant immune modulation, and abnormal epithelialto-mesenchymal transition (EMT) [8–10]. With increasing capacity to synthesize collagen, keloid fibroblasts continuously have high metabolic demands to generate adenosine triphosphate (ATP) [11]. Bayat A et al. documented that fibroblasts derived from the margin of keloids have greater capacity of glycolysis compared with those from central lesions [12]. To overcome energetic consumption, keloid fibroblasts undergo metabolic reprogramming with reduced oxidative phosphorylation and augmented glycolysis [13].

Keloids are regarded as scars with abnormal inflammation [2,14]. Inflammatory responses occur first after cutaneous trauma, after which inflammatory factors trigger subsequent immune response cascades. Interleukins initiate inflammation to regulate the proliferation, apoptosis and differentiation of fibroblasts as well as the extracellular matrix (ECM) production [15,16]. During the early phases, inflammatory factors display a proinflammatory reaction to induce a defence mechanism. In contrast, during the late phases, anti-inflammatory responses overwhelm tissue cell proliferation and remodeling. The occurrence of cytokine imbalance at any stage during wound healing can lead to scar formation [17]. Excessive and prolonged inflammatory responses tend to lead to the development of pathological scars. The inflammation intensity is positively associated to the scar size.

Free calcium ions are important for cellular activities, including cell proliferation, differentiation, movement and death [18,19]. Wang et al. reported that the intracellular free calcium ions could regulate the scar contraction [18]. Moreover, intralesional injection of calcium antagonists can turn on procollagenase gene expression and inhibit VEGF and IL-6 in keloids [20,21]. Boggio et al. and Doong et al. reported that calcium antagonists could alter cell shape and depolymerize actin filaments [22,23]. Activated Ca2^+^-activated K^+^ channels promote cell proliferation, collagen secretion, and contraction in idiopathic pulmonary fibrosis [24]. Therefore, the involvement of calcium ions in keloids might be anticipated.

Phosphoglucose isomerase (PGI), a ubiquitous cytosolic enzyme, controls the second step of glycolysis [25]. It catalyzes the reversible interconversion of glucose-6phosphate into fructose-6 phosphate, which acts as an aldose–ketose isomerase. As a multifunctional enzyme, PGI is involved in gluconeogenesis and the oxidative branch of the pentose phosphate pathway (PPP) [26]. PPP can generate reducing agents against oxidative stress [27]. The overexpression of PGI and its receptor affects glycolysis and promotes the development of cancers such as colorectal cancer and high-grade glioma [27–29]. Moreover, PGI not only maintains glucose metabolism equilibrium but also acts as a cytokine known as autocrine motility factor (AMF) and thereby induces cell proliferation, differentiation, or metastasis [30]. In keloids, an elevated AMF level in tissues has been reported [31], which stimulates fibroblast proliferation and migration.

However, how PGI regulates calcium influx and inflammation in keloids needs clarified.

Moreover, the pathophysiologic role of PGI in keloid pathogenesis remains unsettled.

In the current study, we aimed to elucidate the regulation of inflammatory and fibrotic mechanisms by PGI in keloids.

## 2. Methods

### 2.1. Human samples

Normal skin tissues, excess normal skin next to the resection of original benign lesions such as epidermal cysts or melanocytic nevus, were collected from 6 patients. Keloid tissues, continuous-growth lesions for more than 6 months without local treatments within 3 months, were collected from 6 patients (Table 1). These samples were obtained from participants (Taiwanese people of Han Chinese origin, more than age of 20 years) visiting the Department of Dermatology at Kaohsiung Veterans General Hospital from 2016 to 2019. All patients who donated tissues had signed informed consent and the ethical approval was provided by the Institutional Review Board of Kaohsiung Veterans General Hospital (VGHKS16-CT5-10) according to the relevant regulations and guidelines.

**Table 1.** Demographic information for donor of keloid patients and normal cohorts.

Strains	Age (years)	Gender	Race	Body sites	Duration (years)
Normal 1	32	Female	Han	Elbow	NA
Normal 2	63	Male	Han	Inguinal area	NA
Normal 3	24	Female	Han	Buttock	NA
Normal 4	41	Female	Han	Forearm	NA
Normal 5	45	Male	Han	Cheek	NA
Normal 6	22	Male	Han	Chest	NA
Keloid 1	50	Male	Han	Chest	2 years
Keloid 2	58	Male	Han	Chest	3 years
Keloid 3	44	Female	Han	Chest	2 years
Keloid 4	65	Male	Han	Chest	4 years
Keloid 5	22	Female	Han	Ear	3 years
Keloid 6	26	Female	Han	Shoulder	10 years

NA: not applicable.

### 2.2. Cell culture and treatment

Dermal tissues of normal or keloid skin were cut into 1-2-mm^3^ pieces and digested overnight in DMEM (12100046, Gibco) containing 0.25% trypsin to isolate human fibroblasts. Fibroblasts were cultured with DMEM containing 1% penicillinstreptomycin (15140122, Gibco) and 10% fetal calf serum (FCS) (A4766801, Gibco) at 37 °C in humidified air with 5% CO_2_. Three strains of keloid fibroblasts (KFs) and normal fibroblasts (NFs) derived from tissue samples, within the sixth passage were harvested for the current study. To suppress the activity of PGI fibroblasts were then treated with various concentrations of PGI pharmacological inhibitor, erythrose 4-phosphate (ER4P) (5, 10, 20 and 50 µM) (SI-E0047, Sigma) for 48h [29,30, 32,33]. ER4P was dissolved in double distilled water (ddH_2_O) and then adjusted to the final concentrations.

### 2.3. Migration assay

The scratch wound healing assay was used to determine the migration of fibroblasts. Fibroblasts were seeded on a 12-well plate and cultured to form a confluent monolayer. A 200-µl pipette tip was used to scratch over the well surface to create a vertical line, and the detached cells were washed out with PBS. After scratching, the cell migration was recorded for 0, 4 and 8h by photograph. The ratio of the migrated area to the original area was calculated as the migration rate. 6 randomly selected fields in each well were analyzed.

### 2.4. Immunohistochemistry study

Sections of paraffin-embedded normal skin and keloid tissues were deparaffinized, rehydrated, and received antigen retrieval. Endogenous peroxidise activity was quenched with Peroxidase Block. Excess proteins were blocked with Protein Block

(RE7102, Leica). Sections were applied with 1:100-diluted rabbit anti-human PGI (#57893, Cell Signaling) and 1:500-diluted mouse anti-human vimentin primary antibodies (#5741, Cell Signaling) at 4 °C overnight followed by washing and incubated with secondary antibodies conjugated with 1:500-diluted goat anti-mouse Alexa Fluor^™^ 488 (A11001, Invitrogen) and goat anti-rabbit Alexa Fluor^™^ 568 (A11011, Invitrogen). After incubating with 1:1000-diluted DAPI staining (62248, Thermo Fisher) and Novolink Polymer. sections were counterstained with hematoxylin, dehydrated, and mounted with mounting medium. 6 randomly selected fields in each section were observed under a microscope (Olympus).

### 2.5. Immunofluorescence study

Fibroblasts were seeded into a 12-well plate at a density of 1 × 10^5^ cells/well and incubated overnight. After ER4P treatment, the fibroblasts were fixed by 4% paraformaldehyde, permeabilized by PBS containing 0.1% Triton X-100. The fixed cells were blocked with 10% skim milk without fat in PBS, applied with 1:100-diluted anti- PGI primary antibody (#57893, Cell Signaling) at 4 °C overnight, washed repeatedly with PBS, incubated with secondary antibodies conjugated with 1:500diluted goat anti-rabbit Alexa Fluor^™^ 488 (A11008; Invitrogen), goat anti-mouse Alexa

Fluor^™^ 488 (A11001, Invitrogen) and goat anti-mouse Alexa Fluor^™^ 568 (A11004, Invitrogen). After 1:1000-diluted DAPI staining (62248, Thermo Fisher), sections of stained cells were observed under a fluorescence microscope. The mean intensity of PGI was determined from 5 randomly selected fields each section using ImageJ.

### 2.6. RNA extraction, reverse transcriptase PCR (RT–PCR), and quantitative real-time PCR (qPCR)

TRIzol reagent (15596018, Invitrogen) was used to extract RNA from fibroblasts. Total RNA (1.5 μg) was reverse transcribed into cDNA based on the protocol provided with Superscript III manufacturer (18080085, Invitrogen). The resulting cDNAs were amplified with fast SYBR green PCR Master mix (4385612, Thermo Fisher). Each sample was determined in triplicate on an ABI StepOnePlus Real-Time PCR System (Life Technologies). The β-actin gene was used as an internal reference and PCR amplified with the primer pairs (forward: 5′- GATGAGATTGGCATGGCT TT −3′ and reverse: 5′- GTCACCTTCACCGT CCAGT −3′). The PGI was PCR amplified with the primer pairs (forward: 5′- CCCAGGAGACCATCACGAAT −3′ and reverse: 5′-GCCGCCTGGAGAAACCA −3′). The IL-33 was PCR amplified with the primer pairs (forward: 5′- GGCTGCATCACGTTGTACTTTG −3′ and reverse: 5′- CCACATGTAGATTAGGCCTCAGATT −3′). The NF-κB was PCR amplified with the primer pairs (forward: 5′-AGAAGTGCAGAGGAAACGTCAGA-3′ and reverse: 5′- CTACCACCGCCGAAACTATCC-3′). The TNF-α was PCR amplified with the primer pairs (forward: 5′- CTCTTCTGCCTGCTGCACTTTG −3′ and reverse: 5′- ATGGGCTACAGGCTTGTCACTC −3′). The IL-1 was PCR amplified with the primer pairs (forward: 5′-TTGCCCATCCAAACTTGTTTATT-3′ and reverse: 5′- CCCCCCTGCCAAGCA-3′). The IL-6 was PCR amplified with the primer pairs (forward: 5′-GGTACATCCTCGACGGCATCT-3′ and reverse: 5′-GTGCCTCTTTGCTGCTTTCAC-3′). The transforming growth factor-β (TGF-β) was PCR amplified with the primer pairs (forward: 5′- TACCTGAACCCGTGTTGCTCTC3′ and reverse: 5′- GTTGCTGAGGTATCGCCAGGAA-3′). All primers were manufactured by Genomics (New Taipei City, Taiwan).

### 2.7. Western blotting analysis

Cell Lysis Buffer (ab152163, Abcam) was used to extract cell protein lysate from fibroblasts. Total protein (40 μg/lane) was separated by SDS-PAGE and then electrophoretically transferred onto a PVDF membrane. The membrane was blocked by 5% milk without fat in PBST for 60 min at room temperature. The membrane was applied with primary antibodies by 1:2000-diluted anti- PGI (#57893, Cell Signaling), 1:2000diluted anti-smooth muscle actin (SMA) (14395-1-AP, proteintech) and 1:1000-diluted anti-type I collagen (67288-1-Ig, proteintech) overnight at 4 °C and then incubated with goat-anti-rabbit HRP-conjugated secondary antibody (20202, Leadgene Biomedical). After thoroughly washing, the protein bands were visualized on the membrane using an enhanced chemiluminescence detection system. The 1:5000-diluated β-actin (66009-1-Ig, proteintech) was used as an internal reference.

### 2.8. Quantification of intracellular calcium content

The Calcium Detection Assay Kit (Abcam) was used to determine the intracellular calcium content. Fibroblasts were loaded with 3 mM Fluo-4 and incubated with 5% (v/v) PBX Signal Enhancer and 500 mM probenecid in physiological salt solution for 20 min at 37 °C. Next, the Fluo4-loaded cells were washed with Ca2^+^-free extracellular buffer. To deplete internal calcium stores, 10 µM thapsigarin (TG), sarcoplasmic/endoplasmic reticulum Ca2^+^-ATPase (SERCA) inhibitor, was added. The fluorescence imaging at excitation and emission wavelengths of 488 and 526 nm was recorded using a m confocal laser scanning microscopy (Leica).

### 2.9. Statistical analysis

All values were demonstrated as the mean ± standard deviation (SD) of three independent experiments and analyzed with Prism 6.0 (GraphPad Software). The dataset (GSE220300) of single-cell RNA sequencing (scRNA-seq) was processed, sequenced, integrated into batch-correction using the Harmony algorithm after quality control. The Seurat’s “FindAllMarkers” function was used to identify and annotate cell clusters based on differentially expressed genes. Cell distribution was analyzed by a dimensionality reduction clustering method and projected with the Uniform Manifold Approximation and Projection (UMAP) algorithm. Differences among groups were analyzed using Student’s t test, one-way ANOVA, or the Mann–Whitney test for normally distributed data. The p value less than 0.05 was regarded to indicate a statistically significant difference.

## 3. Results

### 3.1. PGI expression was increased in hyperfibrotic regions in keloid tissues and KFs

**Figure 1.**
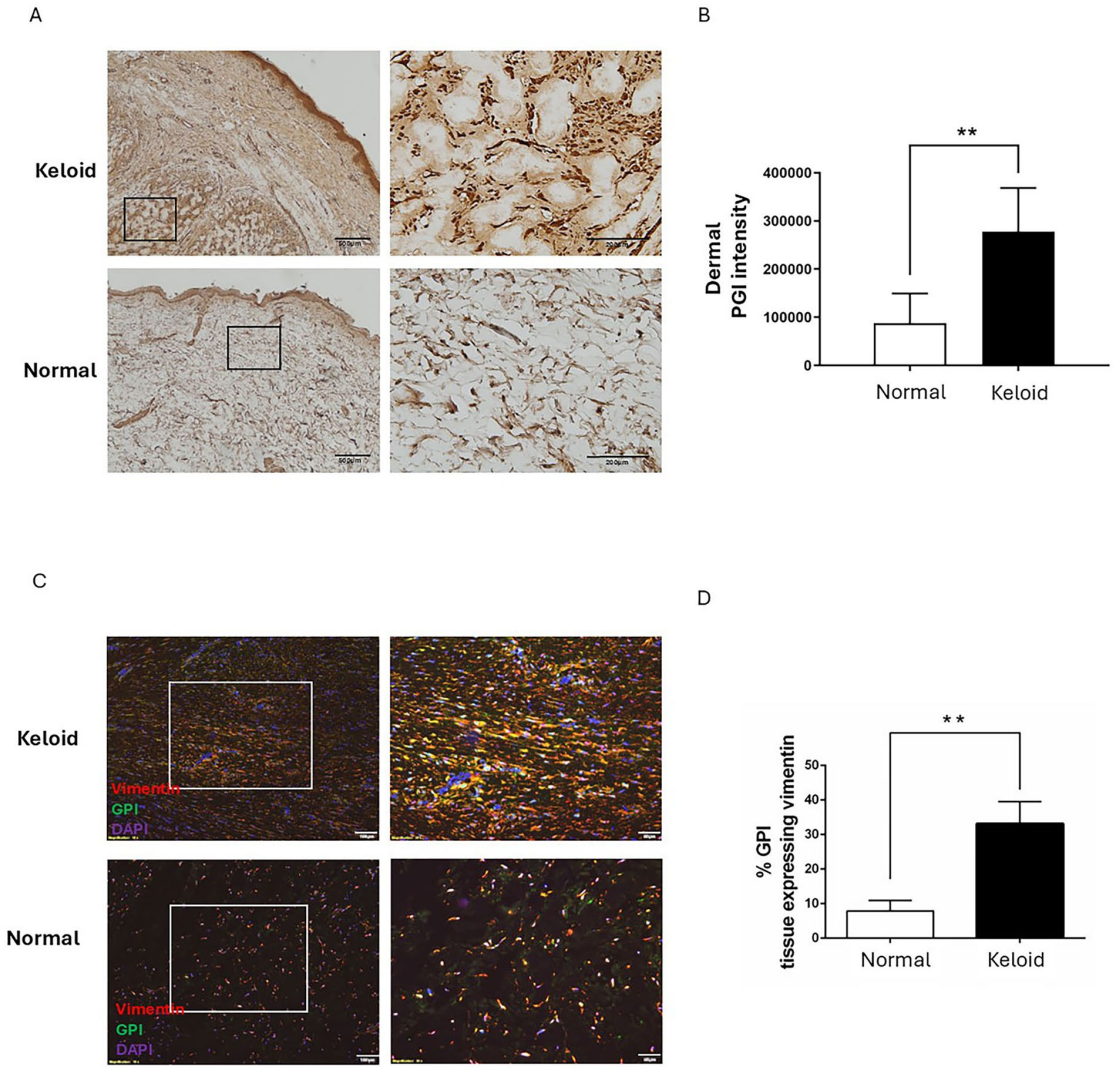
PGI expression was increased in hyperfibrotic regions in keloid tissues. (A) Immunohistochemical staining for PGI in normal skin and keloids (original magnification 40). Right panels are high-power views of the tissues (original magnification 200). The black rectangles in the left panels indicate the areas enlarged at high magnification, respectively. The PGI intensity is shown (B). (C) Sections of keloid tissues and normal skin were deparaffinized and further incubated with antibodies against PGI and vimentin. The expression of PGI (green) and vimentin (red) was localized in keloid tissue and normal skin (original magnification 100). Right panels are high-power views of tissues, identifying colocalization of PGI (green) and vimentin (red) in keloid and normal skin (original magnification 200). The white rectangles in the left panels show the enlarged areas at high magnification, respectively. The number of PGI-positive, vimentin-expressing cells was quantified from five randomly selected high-power fields. The percentage of these cells normalized to total cells (DAPI-stained cells) in the hypercellular areas is shown (D). Data are presented as the mean ± SD. (**p < 0.01) from representative data of 3 independent experiments.

Our recent studies revealed that the RNA level of PGI was found to significantly increase in KFs; therefore, we investigated how the PGI expression is present in keloid tissues [34]. We determined PGI expression in keloid and normal skin tissues using immunohistochemical analysis. The results revealed that PGI was expressed in epidermal tissues from both keloid and normal skin. Interestingly, the dermis of keloids contained greater amounts of PGI and more collagen deposits than normal skin did (Figure 1A-B). Next, we measured the expression levels of PGI and vimentin (as a cytoplasmic marker of fibroblasts) in keloid and normal skin tissues. These results revealed that PGI and vimentin expression levels were also significantly greater in the hypercellular area of keloids than in the dermis of normal skin (Figure 1C). Vimentinpositive cells of keloids displayed higher PGI expression than cells of normal skin did (Figure 1D). These results suggested that the PGI is mostly expressed in fibroblasts and that the level in keloid tissue is greater than that in normal skin.

**Figure 2.**
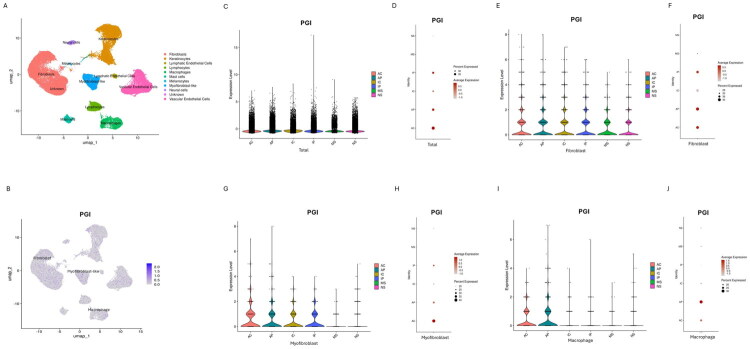
PGI expression was increased in KFs. (A) A UMAP plot of GEO dataset (GSE220300) depicting scRNA-seq of keloids and scars. (B) The UMAP plot showing *PGI* gene is mainly expressed in fibroblasts, myofibroblasts and macrophages. (C-D) The expression of *PGI* gene in keloids was higher than that in mature and normal scars. Notably, *PGI* gene was highest in central region of active keloids. (E) In fibroblasts, violin and (F) dot plots showing the expression of *PGI* gene in active keloids were highest among all the group. (G) In myofibroblasts, violin and (H) dot plots showing the expression of *PGI* gene in active keloids were highest among all the groups. (I) In macrophages, violin and (J) dot plots showing the expression of *PGI* gene in active keloids were highest among all the group. AC, center region of active keloids; AP, peripheral region of active keloids; IC, center region of inactive keloids; IP, peripheral region of inactive keloids; MS, mature scar; NS, normal scar.

To validate the PGI expression in keloids by analyzing the public scRNA-seq dataset GSE220300, our results revealed that PGI was predominantly expressed in fibroblasts, myofibroblasts and macrophages (Figure 2A-B). Compared to mature and normal scars, keloids exhibited significantly higher expression of *PGI* gene at the tissue level (Figure 2C), in which the highest PGI expressions were localized to the center region of active keloids (Figure 2D). This pattern was particularly pronounced specifically within fibroblasts (Figure 2E-F), myofibroblasts (Figure 2G-H) and macrophages (Figure 2I-J).

### 3.2. PGI controls fibrotic activity in KFs

**Figure 3.**
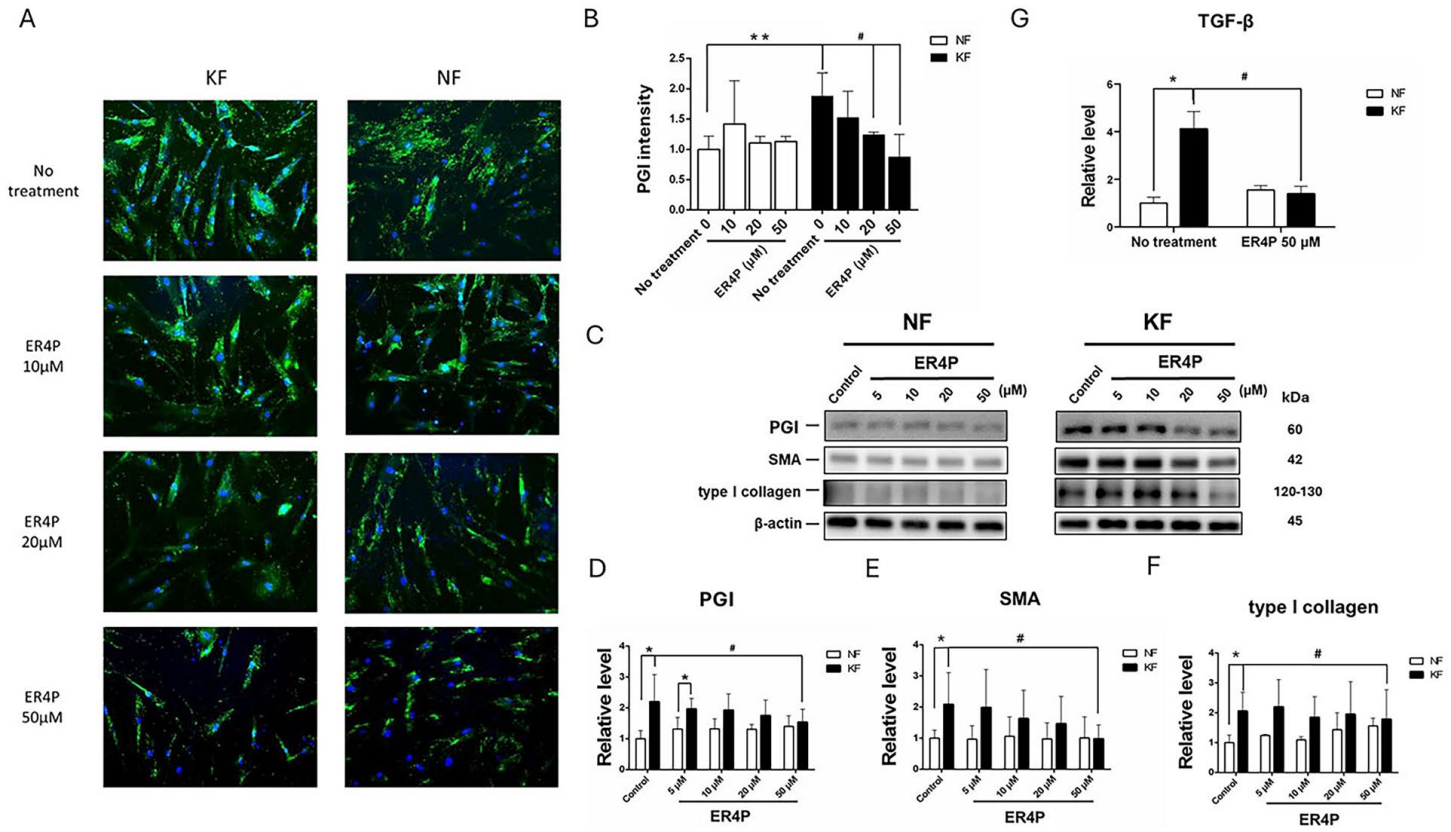
PGI was involved in fibrotic activity in KFs. NFs and KFs pretreated with or without the indicated dose of ER4P for 48 h (A) Immunofluorescence staining for PGI in NFs and KFs (original magnification 200). (B) Quantification of IF form (A). Five random fields of each section were selected, and the PGI intensity was measured. (C) Representative bands and quantification of (D) PGI, (E) SMA and (F) type I collagen in NFs and KFs determined by western blot examination. The β-actin level was used as a loading control. (G) NFs and KFs were pretreated with or without 50 µM ER4P for 48 h. RNA was purified, and the mRNA expression levels of *TGF-β* were determined by RT–PCR. Data are presented as the mean ± SEM. (*p < 0.05, **p < 0.01 NFs vs. KFs; ^#^*p* < 0.05 KFs baseline vs. KFs receiving indicated dose of ER4P) from representative data of 3 independent experiments.

In parallel, PGI expression in fibroblasts was measured by immunofluorescence staining. The results revealed that PGI expression was significantly greater in KFs than in NFs (Figure 3A). Treatment of KFs with 20 or 50 µM ER4P substantially decreased the PGI level in KFs (Figure 3B).

Keloids are characterized by fibroproliferative disorders, in which TGF-β induces the synthesis of α-SMA and collagen in fibroblasts. Therefore, we measured the protein levels of PGI, SMA and type I collagen, which were significantly greater in KFs than in NFs. Treatment with ER4P decreased the levels of PGI (Figure 3C-D), SMA (Figure 3C, E) and type I collagen in KFs (Figure 3C, F). Since TGF-β emerges as a potent profibrotic factor, we measured the RNA level, which was significantly greater in KFs than in NFs. Treatment with 50 µM ER4P for 48 h decreased the *TGF-β* level in KFs (Figure 3G). Taken together, these data suggest that the PGI may contribute to the fibrotic lesions’ formation in keloids.

### 3.3. PGI regulated inflammatory activity in KFs

**Figure 4.**
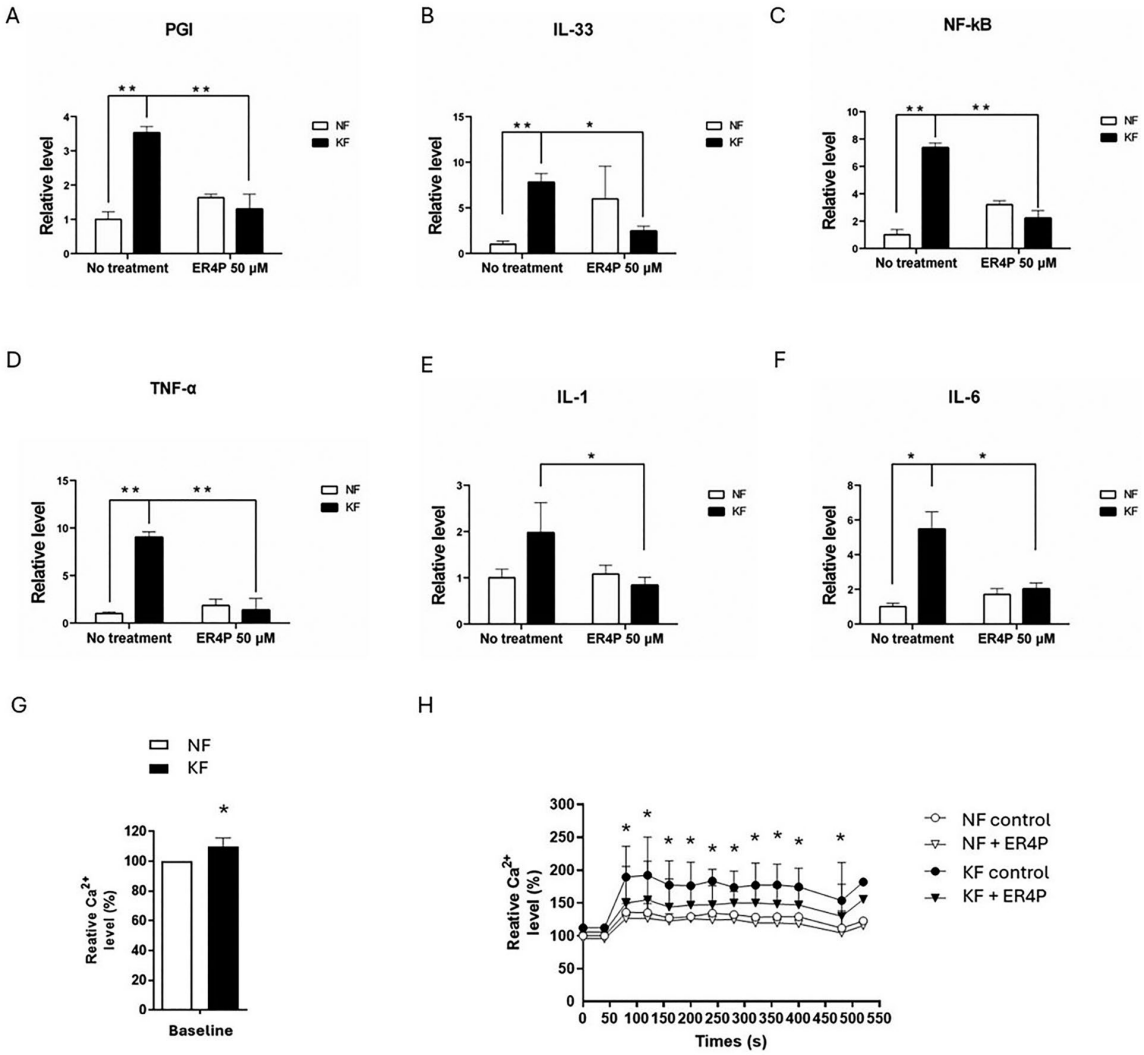
Blocking PGI decreased the activation of proinflammatory cytokines and enhanced cytosolic Ca2+ signaling in KFs. NFs and KFs were pretreated with or without 50 µM ER4P for 48 h. RNA was purified, and the mRNA expression levels of proinflammatory factors were determined by RT–PCR. The mRNA expression levels of (A) *PGI*, (B) *IL-33*, (C) *NK-κB*, (D) *TNF-α*, (E) *IL-1* and (F) *IL-6* in KFs and NFs. Data are presented as the mean ± SD. (*p < 0.05, **p < 0.01) from representative data of 3 independent experiments. (G) The baseline calcium content was measured using a calcium detection assay kit. (H) The cytosolic Ca2^+^ signals in NFs and KFs after treatment with 50 µM ER4P were assessed by Fluo-4 Ca2+ measurement. Average peaks of cytosolic Ca2^+^ were detected in control and ERI4P-treated fibroblasts with TG. Data are presented as the mean ± SD. (*p < 0.05 KFs vs. KFs + ER4P) from representative data of 3 independent experiments.

Because keloids are a chronic inflammatory skin disease, we measured proinflammatory factors in KFs and NFs by RT–PCR. The RNA levels of *PGI* (Figure 4A), *IL-33* (Figure 4B), *NK-κB* (Figure 4C), *TNF-α* (Figure 4D), *IL-1* (Figure 4E) and *IL-6* (Figure 4F) were significantly greater in KFs than in NFs. To investigate whether the expression of proinflammatory factors was regulated by PGI, we examined their expression in NFs and KFs treated with 50 µM ER4P for 48h (Figure 4A). These results showed that PGI attenuated the expression of proinflammatory factors in KFs (Figure 4B-F), which suggested that PGI may be involved in the inflammatory pathway in keloids.

### 3.4. Blocking PGI decreased enhanced cytosolic Ca2+ signaling in KFs

The total calcium content in KFs was greater than that in NFs. To investigate whether PGI alter the store-operated calcium influx in fibroblasts, TG was used to deplete Ca2^+^ stores. TG produced a much greater increase in cytosolic free Ca2^+^ levels in KFs than in NFs, indicating that more Ca2^+^ was released from the intracellular Ca2^+^ stores in KFs. Treatment with ER4P decreased the cytosolic free Ca2^+^ levels in KFs but not in NFs (Figure 4G, H). These findings suggest that PGI might be involved in the regulation of intracellular calcium signaling in keloid fibroblasts.

### 3.5. Blocking PGI inhibits fibroblast migration in KFs

**Figure 5.**
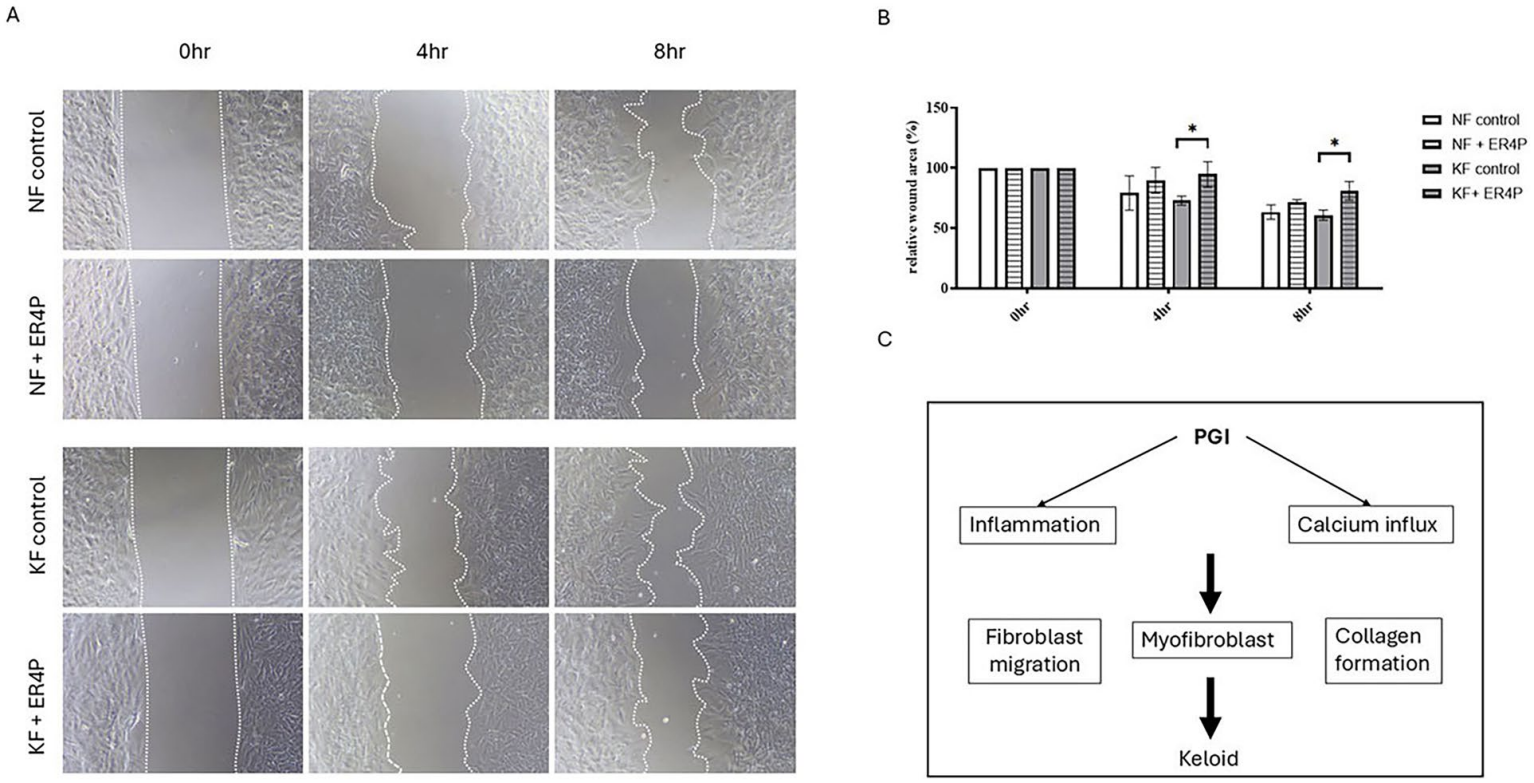
Blocking PGI decreased the migration activity in KFs. NFs and KFs were pretreated with or without 50 µM ERI4P for 48 h. (A) A scratch assay was used to determine cell migration. Representative images (original magnification 200) and (B) quantification of scratch wound healing assay. Data are presented as the mean ± SEM. (*p < 0.05 NFs vs. KFs; ^#^*p* < 0.05 KFs vs. KFs + ER4P) from representative data of 3 independent experiments. (C) Schematic illustrating how PGI contributes to the pathogenesis of keloids.

To identify whether the migration of KFs is regulated by PGI, we evaluated the effects of PGI on keloid cell migration after ER4P treatment by measuring the scratch wound healing assay. At baseline, compared with NFs, KFs had a slightly faster closure rate. In both KFs and NFs, ER4P treatment constrained fibroblast migration, particularly in keloids (Figure 5A-B).

## 4. Discussion

Keloids are regarded as an inflammatory skin disease with altered metabolic demands [34]. Fibroblasts, the main proliferative cells involved in the deposition of collagen fibers and ECM, are the main cells that induce keloid formation [35]. The fibroblasts migrate from the original growth site to normal skin so that keloids grow without self-limitation [36]. We previously reported that glycolytic enzymes, including glucose transporter 1, hexokinase, PGI, and aldolase, were upregulated in KFs compared with their expression levels in NFs, indicating that the induction of these enzymes may contribute to the high energy demands in KFs [34]. The results of the current study provide evidence that the expression of PGI was increased in both hyperfibrotic regions of keloid tissues and KFs. Inhibiting PGI activity can suppress fibroblast migration through ER4P. Moreover, PGI can regulate inflammation and calcium influx in KFs. These novel findings suggest that PGI can control the fibrotic activity in KFs by regulating inflammatory activity and calcium influx. (Figure 5C)

As a potent growth factor during wound healing and fibrosis process, the elevated TGF-β1 in keloid tissues stimulates dermal fibroblast proliferation, differentiation, and collagen formation [37–40]. The differentiation of fibroblasts to myofibroblasts, characterized by the synthesis of SMA, is crucial in the fibrotic process [41]. A previous study revealed that TGF-β1 induced cell rigidity and altered cell mechanical properties through the upregulation of SMA in keloids [1]. Otherwise, TGF-β1 stimulates the release of connective tissue growth factor, thus increasing the production of collagen types I and III in keloids [42]. Therefore, targeting the activity of pro-fibrotic myofibroblast has the potential to slow or halt the keloids’ progression. Our studies also revealed that the RNA level of SMA and the protein levels of SMA and type 1 collagen increased in KFs, confirming that KFs exhibit greater constitutive profibrotic activity.

PGI not only regulates glucose metabolism inside cells but also exhibits multifunctional growth factor-like activity in various extracellular processes [43,44]. PGI overexpression is related to the cancer metastasis and progression in a wide spectrum of malignancies [45,46]. It can prevent tumor cells from stress-induced cell senescence and apoptosis [44,47]. In the fibroblast-like synoviocytes of rheumatoid arthritis, PGI stimulates their proliferation and inhibits their apoptosis with increased secretion of inflammatory cytokines [48]. Silencing of PGI in human fibrosarcoma HT1080 cells inhibited their migration and invasion abilities. Suppression of exogenous PGI also ceased cell proliferation by stopping DNA synthesis [39]. PGI overexpression in NIH-3T3 cells enhances the tumorigenicity and the survival of cells, through PI3K/Akt signaling [47]. In the current study, compared with those in NFs, the PGI expression in KFs was significantly greater, and the fibrotic activity and migration ability of KFs were stronger than those in NFs; in addition, E4RP effectively decreased the fibrotic activity and the migration ability of KFs by inhibiting PGI. Hence, PGI is expected to be a potential target for the treatment of keloids.

Inflammatory responses play key roles as mediators of keloid progression. Patients with keloids have significantly higher cytokine levels than nonkeloid-forming patients do [49]. The increase of proinflammatory cytokines, such as TNF-α, IL-1 and IL-6, in keloid tissue, indicated that localized inflammatory response is present in patients with keloids [14]. As a key proinflammatory cytokine, IL-6 upregulation in KFs enhances an increase in the expression of downstream target genes regarding cell proliferation and the synthesis of matrix, which trigger subsequent collagen fibers production [50]. Other cytokines, such as IL33, IL-13, IL-10 and IL-4 have also been noticed increased in keloids [51–53]. However, their associations in keloids are not well understood. Our studies revealed that the RNA levels of IL-33, NK-κB, TNF-α, IL-1 and IL-6 increased in KFs, confirming that KFs exhibit enhanced proinflammatory activity. Moreover, we demonstrated that ER4P effectively decreased inflammatory activity by inhibiting PGI.

These findings further expand the importance of PGI in keloids.

The KFs in the current study showed a PGI-mediated induction of calcium influx. Calcium is an essential second messenger that regulates a number of physiological processes, such as cell migration, angiogenesis, barrier function, and inflammation [54]. As a key regulator of cell survival, a sustained increase in cytosolic calcium released from the ER could lead to the mitochondrial calcium overload and the release of apoptotic signals [55]. The intracellular Ca2^+^ level is maintained at a very low concentration under normal conditions, whereas the increased level triggers downstream signaling cascades. In endothelial cells, calcium signaling can be stimulated by inflammatory mediators [54]. As a sarco/endoplasmic reticulum Ca2^+^ATPase (SERCA) inhibitor, TG can prevent ER Ca2^+^ uptake to disrupt the tubular calnexin-labelled ER network. In response to TG stimulation, PGI treatment attenuated

TG-induced changes in cytosolic calcium levels and protected against apoptosis in HEK293 cells [56]. In idiopathic pulmonary fibrosis-derived fibroblasts, Ca2^+^activated KCa3.1 potassium channels promoted TGFβ1-dependent profibrotic responses [57]. Compared with NFs, KFs exhibited more single calcium spikes during cyclical stretching [58]. By mechanosensitive channels, cyclical stretching induced calcium spikes in dermal fibroblasts while a Ca2^+^ channel blocker suppressed calcium spikes [59]. Our current study revealed that basal intracellular calcium levels were increased in KFs, which was consistent with the findings of a previous study. After ER4P treatment, the suppression of PGI decreased intracellular calcium levels in accordance with decreased fibrotic activity and fibroblast migration. Altogether, these results suggest that the inhibition of PGI may regulate intracellular calcium influx and coordinate the inflammatory response in keloids.

## 5. Conclusions

In summary, we found that PGI expression was upregulated in both KFs and keloid tissue. PGI controls SMA and type I collagen expression, and cell migration, in KFs. Indeed, PGI regulates inflammation and calcium influx in KFs, indicating the functional effects of PGI on the pathogenesis of fibrosis. This study, for the first time, demonstrated that blocking PGI might be a therapeutic target in keloids through a calcium influx-dependent inflammatory response.

## Data Availability

All data generated or analyzed during this study are included in this published article.
